# Effect of Acid-Labile Protecting-Group Structure on the Micrometer-Scale Patterning Performance of Core-First RAFT-Synthesized Three-Arm Photoresists

**DOI:** 10.3390/polym18182223

**Published:** 2026-09-12

**Authors:** Jinyoung Kim, Yura Choi, Eunsu Park, Kyeongjin Jang, Hyungjun Noh, Namchul Cho

**Affiliations:** Department of Energy Engineering, Soonchunhyang University, 22 Soonchunhyang-ro, Asan 31538, Republic of Korea; thn05007@gmail.com (J.K.); bnbn3237@gmail.com (Y.C.); eunsu0903@sch.ac.kr (E.P.); 0711wkdrudwls@sch.ac.kr (K.J.); 2003gudwns@sch.ac.kr (H.N.)

**Keywords:** three-arm star polymer, RAFT polymerization, chemically amplified photoresist, acid-labile protecting group, negative-tone imaging (NTI), micrometer-scale patterning, critical-dimension fidelity, line-edge roughness (LER)

## Abstract

Controlling dimensional fidelity and line-edge roughness is important in chemically amplified negative-tone imaging. In this study, two three-arm random terpolymers containing different acid-labile protecting groups were synthesized via core-first reversible addition–fragmentation chain-transfer (RAFT) polymerization to examine the influence of protecting-group structure within a common star-polymer framework. HTT 334 contained tert-butyl methacrylate (t-BMA), whereas HTM 334 contained 2-methyl-2-adamantyl methacrylate (MAMA). The two polymers exhibited comparable molecular weights and compositions, with narrow dispersities of 1.14 and 1.16. Thermal analysis showed that HTM 334 had a slightly higher glass-transition temperature than HTT 334 (77 and 73 °C, respectively). Fourier transform infrared (FT-IR) spectroscopy revealed spectral changes after exposure and post-exposure baking (PEB) that were consistent with acid-catalyzed deprotection and possible intermolecular reactions involving the hydroxyethyl methacrylate units. Negative-tone patterns were formed using an n-butyl acetate developer under the selected PEB conditions of 70 °C for HTT 334 and 90 °C for HTM 334. Across nominal mask dimensions of 10–80 μm, HTM 334 exhibited printed-to-mask critical-dimension ratios of 0.990–1.039, compared with 0.915–1.003 for HTT 334. HTM 334 also showed lower measured line-edge roughness values of 0.22–0.39 μm than HTT 334, which showed values of 0.52–1.27 μm. At the nominal 10 μm dimension, HTM 334 exhibited a line-edge roughness of 0.27 μm and an LER/CD ratio of 2.60%. These results indicate that an acid-labile protecting-group structure can influence thermal behavior and micrometer-scale pattern fidelity within the investigated three-arm RAFT polymer platform.

## 1. Introduction

Securing both high-resolution capabilities and pristine pattern quality presents a critical challenge in advanced semiconductor lithography [1,2,3,4,5,6]. Conventional chemically amplified resists (CARs) use linear polymers, which often have long chain lengths and interchain entanglement [7,8]. During development, this entanglement can cause uneven dissolution behavior, causing the polymer to detach in larger aggregate units rather than dissolving at the molecular level, which can increase surface roughness (LER) and leave unexposed residue [9,10,11]. Negative-Tone Imaging (NTI) using organic solvent developers, such as *n*-butyl acetate (*n*-BA), has been introduced as an alternative patterning approach to address the swelling and pattern collapse associated with traditional aqueous alkaline developers [12,13,14,15,16]. Due to the lower polarity of *n*-BA, the unexposed photoresist can dissolve while leaving the exposed regions intact with reduced swelling, which helps improve pattern fidelity. Controlling photoacid diffusion and maintaining the precise dissolution contrast of the resist to optimize MEEF and prevent LER at fine resolutions remain important considerations [17,18,19,20,21].

To address entanglement-related limitations and lower LER, three-dimensional branched architectures, such as star polymers, present an approach that can reduce intermolecular chain entanglement [22,23]. Compared with linear polymers, star polymers have been reported to exhibit more compact conformations and reduced intermolecular chain entanglement owing to their branched architecture [22,23,24,25,26,27]. Such compact branched architectures have been proposed to reduce interchain entanglement and promote more uniform dissolution, which has been suggested to be beneficial for dissolution behavior during organic-solvent development. Recent advances in RAFT polymerization have further expanded its utility for the controlled synthesis of complex polymer architectures and light-mediated polymerization processes [28,29].

To maintain thermal stability and improve dissolution contrast in the organic developer, adjusting the steric bulk of the acid-labile protecting group can be considered [30,31,32]. Multi-arm star polymers often have a lower glass transition temperature (T*_g_*) due to an increased free volume from multiple chain ends, which can make them susceptible to critical dimension (CD) variations during the post-exposure bake (PEB). Incorporating bulky cycloaliphatic groups, such as the 2-methyl-2-adamantyl methacrylate (MAMA) moiety, can restrict polymer chain mobility and increase the overall T*_g_*, contributing to the mechanical strength [33,34,35]. Additionally, the hydrophobicity and organic affinity of the adamantyl groups can improve the solubility of the unexposed polymer in the *n*-BA developer, supporting dissolution and contrast in the NTI process [36,37].

This study compares two core-first RAFT-synthesized 3-arm random terpolymers containing different acid-labile protecting-group units: tert-butyl methacrylate (t-BMA, HTT 334) and 2-methyl-2-adamantyl methacrylate (MAMA, HTM 334). The two polymers exhibited comparable molecular weights and monomer compositions, enabling the influence of protecting-group structure to be examined within the same 3-arm polymer framework. Their thermal behavior, FT-IR spectral changes after exposure and post-exposure baking, and micrometer-scale negative-tone patterning performance in an *n*-butyl acetate developer were evaluated. Patterning performance was assessed using printed critical dimensions, printed-to-mask CD ratios, and line-edge roughness under the selected PEB condition for each polymer. The observed differences are discussed in relation to protecting-group structure, thermal response, reaction kinetics, and developer interaction, while acid diffusion, crosslink density, and the effect of star topology relative to a linear analogue were not directly determined in this study. Previous studies have demonstrated the synthesis and lithographic applicability of star-shaped photoresists; however, systematic comparisons of acid-labile protecting-group chemistry within a common three-arm RAFT polymer architecture remain limited. Therefore, the present study specifically examines how the chemical structure of the acid-labile protecting group affects patterning behavior within a comparable three-arm RAFT polymer architecture.

## 2. Materials and Methods

### 2.1. Materials

The base methacrylate monomers, including 2-hydroxyethyl methacrylate (HEMA), tetrahydrodicyclopentadienyl methacrylate (TCDMA), and *tert*-butyl methacrylate (*t*-BMA), were purchased from Tokyo Chemical Industry (Tokyo, Japan), and 2-methyl-2-adamantyl methacrylate (MAMA) was purchased from Sigma-Aldrich (St. Louis, MO, USA). For the synthesis of the 3-armed chain transfer agent (CTA), trimethylolpropane (TMP), *N,N′*-dicyclohexylcarbodiimide (DCC), and 4-dimethylaminopyridine (DMAP) were purchased from Sigma-Aldrich (St. Louis, MO, USA). 2,2’-Azobis(2,4-dimethylvaleronitrile) (V-65) was purchased from FUJIFILM Wako Pure Chemical Corporation (Osaka, Japan). The RAFT agent precursor, 4-cyano-4-(dodecylsulfanylthiocarbonyl)sulfanylpentanoic acid (CEPA), was purchased from BLD Pharm(Shanghai, China). The photoacid generator (PAG), *N*-((trifluoromethylsulfonyl)oxy)-5-norbornene-2,3-dicarboximide (NDI), was purchased from Alfa Aesar (Haverhill, MA, USA). Propylene glycol monomethyl ether acetate (PGMEA) was purchased from Sigma-Aldrich (St. Louis, MO, USA). Diethylene glycol monoethyl ether (DGME), *n*-butyl acetate, and *n*-hexane were purchased from DUKSAN (Seoul, Republic of Korea). All reagents and solvents were used without further purification unless otherwise specified.

### 2.2. Synthesis of 3-Armed Chain Transfer Agent (TMP-CEPA_3_)

The 3-armed CTA (TMP-CEPA_3_) was synthesized via an esterification reaction connecting a central core to three RAFT agent functionalities. First, the core molecule, trimethylolpropane (TMP, 0.40 g, 2.98 mmol), the RAFT precursor, 4-cyano-4-(dodecylsulfanylthiocarbonyl)sulfanylpentanoic acid (CEPA, 2.82 g, 10.72 mmol), and the catalyst, 4-dimethylaminopyridine (DMAP, 0.11 g, 0.89 mmol), were introduced into a dried jacketed reactor. The reactor was thoroughly deoxygenated and maintained under anhydrous conditions by performing three consecutive vacuum–nitrogen purging cycles, after which the system was cooled to 0 °C. Anhydrous tetrahydrofuran (THF, 100 mL) was then added to the reactor, and the mixture was stirred for 30 min. Subsequently, a coupling agent solution containing *N,N′*-dicyclohexylcarbodiimide (DCC, 2.76 g, 13.42 mmol) dissolved in 20 mL of dichloromethane (DCM) was added dropwise to the reaction mixture at 0 °C. During this addition, a white precipitate of dicyclohexylurea (DCU) was formed as a byproduct. To complete the reaction, the temperature was gradually raised to 25 °C, and the mixture was allowed to continuously react for 48 h. After the reaction was completed, the white precipitate was removed by filtration, and the volatile solvents (THF and DCM) were evaporated under reduced pressure. The obtained crude product was redissolved in DCM and washed sequentially with a 0.5 N aqueous HCl solution and a saturated aqueous NaHCO_3_ solution to completely remove any residual basic compounds. Finally, the DCM layer was evaporated, and the crude material was purified by silica gel column chromatography using a mixture of ethyl acetate and *n*-hexane (1:8) as the eluent, yielding the highly hydrophobic target product, TMP-CEPA_3_.

### 2.3. Preparation of 3-Armed Methacrylate Polymers

The 3-armed methacrylate polymers containing different protecting groups (MAMA or *t*-BMA) were synthesized via RAFT polymerization utilizing the newly synthesized TMP-CEPA_3_ as the chain transfer agent. The synthesis was carried out using a simultaneous monomer addition approach to form random terpolymer arms originating from the core. First, the specific monomers—2-hydroxyethyl methacrylate (HEMA), tetrahydrodicyclopentadienyl methacrylate (TCDMA), and the target protecting monomers (either MAMA or t-BMA)—were added to a jacketed reactor at a molar feed ratio of 3:3:4, respectively. The nominal total monomer-to-TMP-CEPA_3_ molar ratio was approximately 760:1. A separate initiator solution was prepared by dissolving V-65 (0.02 g) and TMP-CEPA_3_ (0.20 g) in propylene glycol monomethyl ether acetate (PGMEA). The amount of PGMEA solvent was adjusted to 30 wt% relative to the total mass of the loaded monomers. To ensure an inert environment, the jacketed reactor was subjected to three vacuum–nitrogen purging cycles under dry conditions. The V-65/CTA solution was then injected into the reactor containing the monomers. The RAFT polymerization was initiated by heating the reactor to 60 °C, and the mixture was continuously stirred for 20 h. Once the polymerization was complete, the reaction was immediately terminated by immersing the flask in an ice-water bath to rapidly quench the generated radicals. The quenched polymer solution was then added dropwise into a large excess of distilled water (approximately 10 times the volume of the PGMEA used) to induce precipitation. The precipitated solid was collected and dried thoroughly in a vacuum oven to afford the final 3-armed photoresist polymers in powder form.

### 2.4. Preparation of 3-Armed Photoresist-Based Film

The photoresist solutions were prepared by dissolving 0.5 g of the synthesized 3-arm polymers (HTT 334 or HTM 334) and 25 mg of NDI as the PAG in a mixed solvent of PGMEA and DGME at a volume ratio of 7:3. The mixture was stirred for 4 h at room temperature to ensure complete dissolution. To prepare the patterns, the photoresist solution was spin-coated onto a bare 1 × 1 cm silicon wafer at 1000 rpm for 1 min. After coating, the photoresist films were soft-baked at 80 °C for 1 min on a hot plate to evaporate the residual solvent. For the exposure step, the photomask was aligned with the baked wafers, and the films were exposed to a 365 nm UV light source at a dose of 30 mJ/cm^2^. Subsequently, post-exposure baking (PEB) was conducted for 1 min at 70 °C for the HTT 334 polymer and 90 °C for the HTM 334 polymer. Finally, the annealed wafers were developed by dipping them in *n*-butyl acetate for 10 s, followed by washing with *n*-hexane. The patterned films were then hard-baked at 80 °C for 1 min.

### 2.5. Characterization

Proton nuclear magnetic resonance (^1^H NMR) measurements were performed using a 400 MHz Ascend 400 spectrometer (Bruker Corporation, Billerica, MA, USA) with CDCl_3_ as the solvent and 64 scans. Molecular weight distributions were determined by gel permeation chromatography (GPC, Waters, Worcester County, MA, USA) using THF as the mobile phase. The mobile phase flow rate was set to 0.1 mL/min, and the GPC system was equipped (Alliance 2695, Waters Corporation, Milford, MA, USA) with KS-801, KS-802, and KS-803 columns, along with a differential refractive index (RI 804) detector (Agilent Technologies, Santa Clara, CA, USA). The glass transition temperatures (T*_g_*) of the polymers were determined by means of differential scanning calorimetry (DSC, Q20 from TA Instruments Ltd., New Castle, DE, USA) in the range of 40–200 °C at a heating rate of 5 °C per minute under a nitrogen atmosphere. Thermogravimetric analysis (TGA, Q50, TA Instruments Ltd., New Castle, DE, USA) was performed from 30 to 400 °C at a heating rate of 10 °C min^−1^ under a nitrogen atmosphere. The temperature at 5% mass loss (T_5_%) was defined as the temperature at which the residual weight reached 95% of the initial sample weight. FT–IR spectra were recorded using a FT-200 spectrophotometer (Horiba, Kyoto, Japan). Pattern images were captured using an optical microscope (BX53M, OLYMPUS, Shinjuku, Japan) at various magnifications.

## 3. Results and Discussion

### 3.1. Synthesis of 3-Armed Star Polymers Through Core-First RAFT Polymerization

**Scheme 1:**
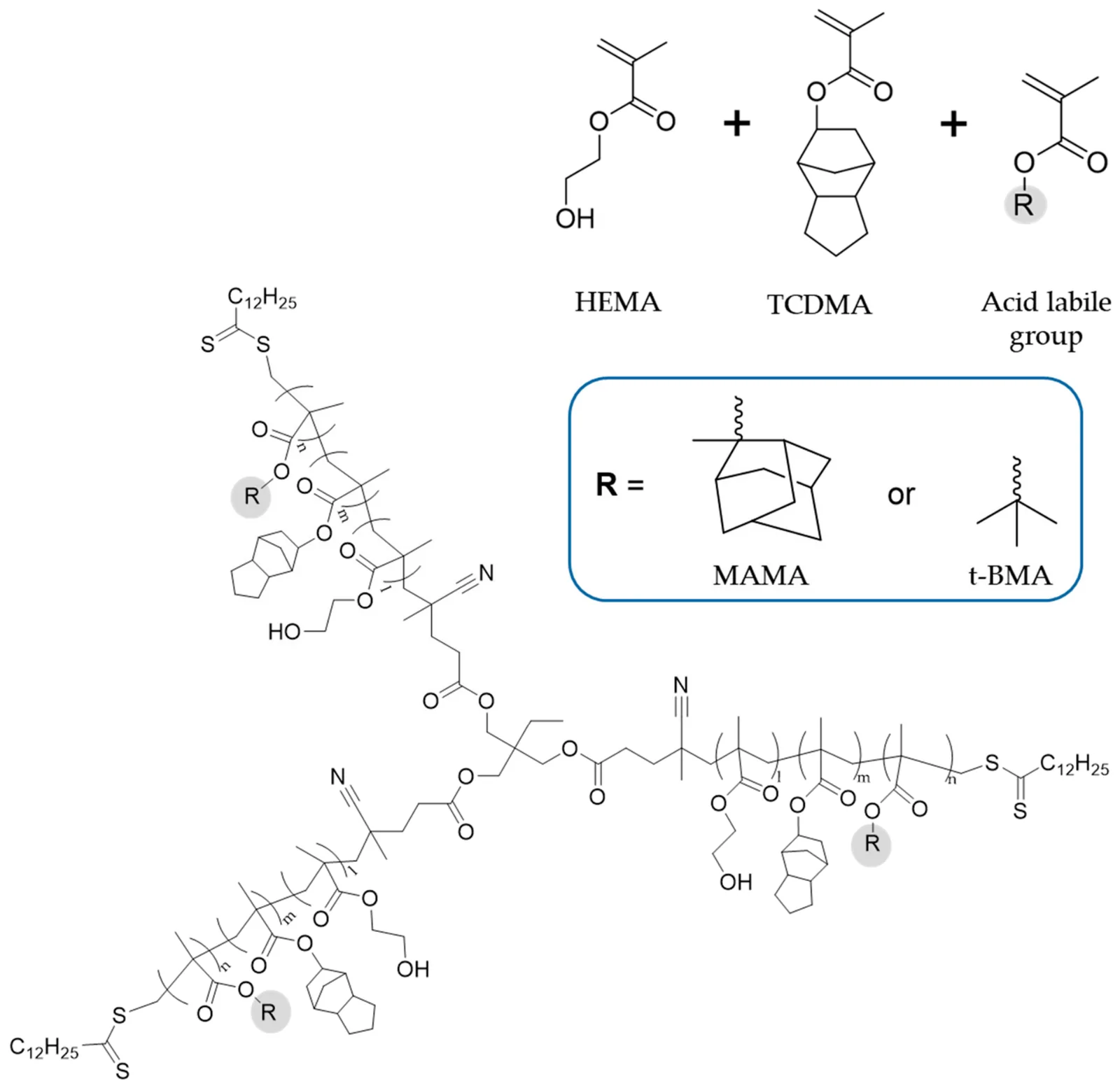
Chemical structure of the synthesized 3-arm star-shaped photoresist polymer centered on the chain transfer agent (CTA). The polymer chain incorporates each monomer unit: HEMA, TCDMA, and an acid-labile protecting group denoted as R, where R represents either the MAMA (HTM 334) or *t*-BMA (HTT 334) monomer.

To investigate the effect of protecting group steric hindrance on the patterning performance in the negative-tone imaging (NTI) process, two structurally distinct 3-arm random terpolymers were synthesized via reversible addition–fragmentation chain transfer (RAFT) polymerization [22,25]. As illustrated in Scheme 1, a “core-first” approach was employed utilizing a 3-arm chain transfer agent (TMP-CEPA3) [25]. The base polymer matrix consists of HEMA, which provides affinity to the developer and substrate adhesion [30], and TCDMA, which imparts plasma etch resistance [35]. For the acid-labile protecting groups, two monomers with different steric bulk were selected: the bulky MAMA (HTM 334) and the relatively flexible *t*-BMA (HTT 334) [33,38].

**Figure 1:**
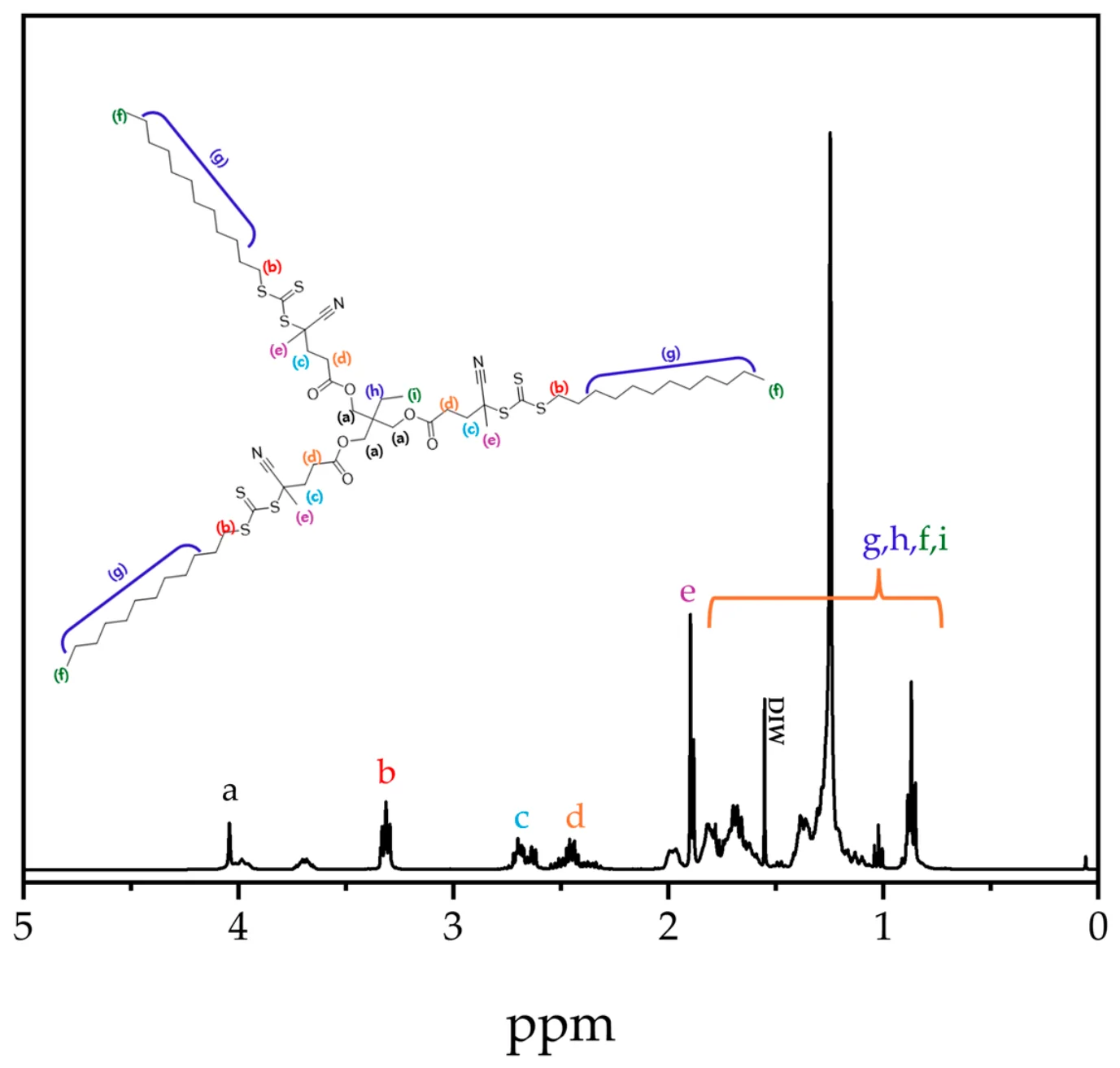
^1^H NMR spectrum of the synthesized 3-arm CTA (TMP-CEPA_3_) in CDCl_3_. The colored labels (a–i) correspond to the proton assignments indicated in the chemical structure.

**Figure 2:**
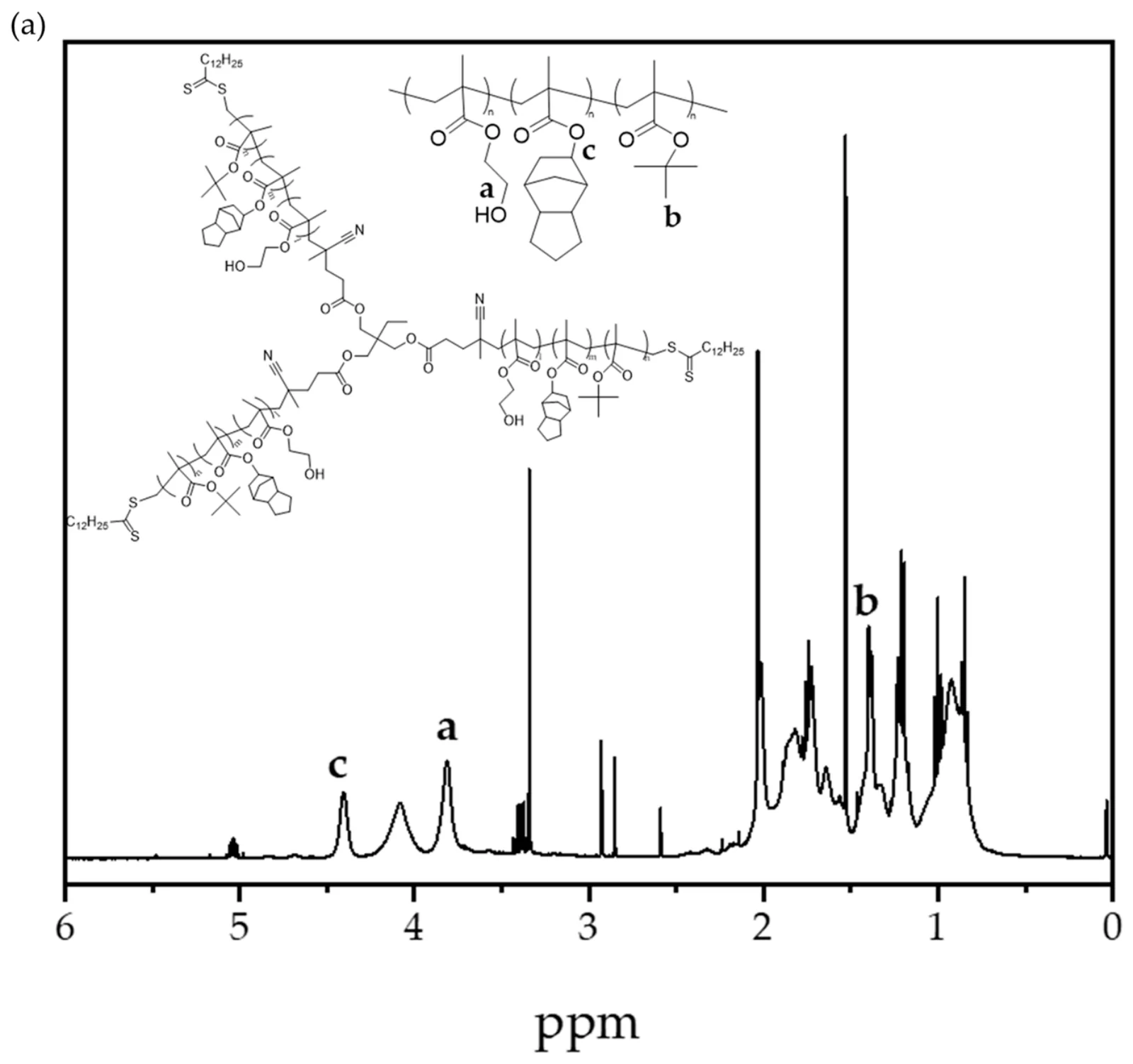

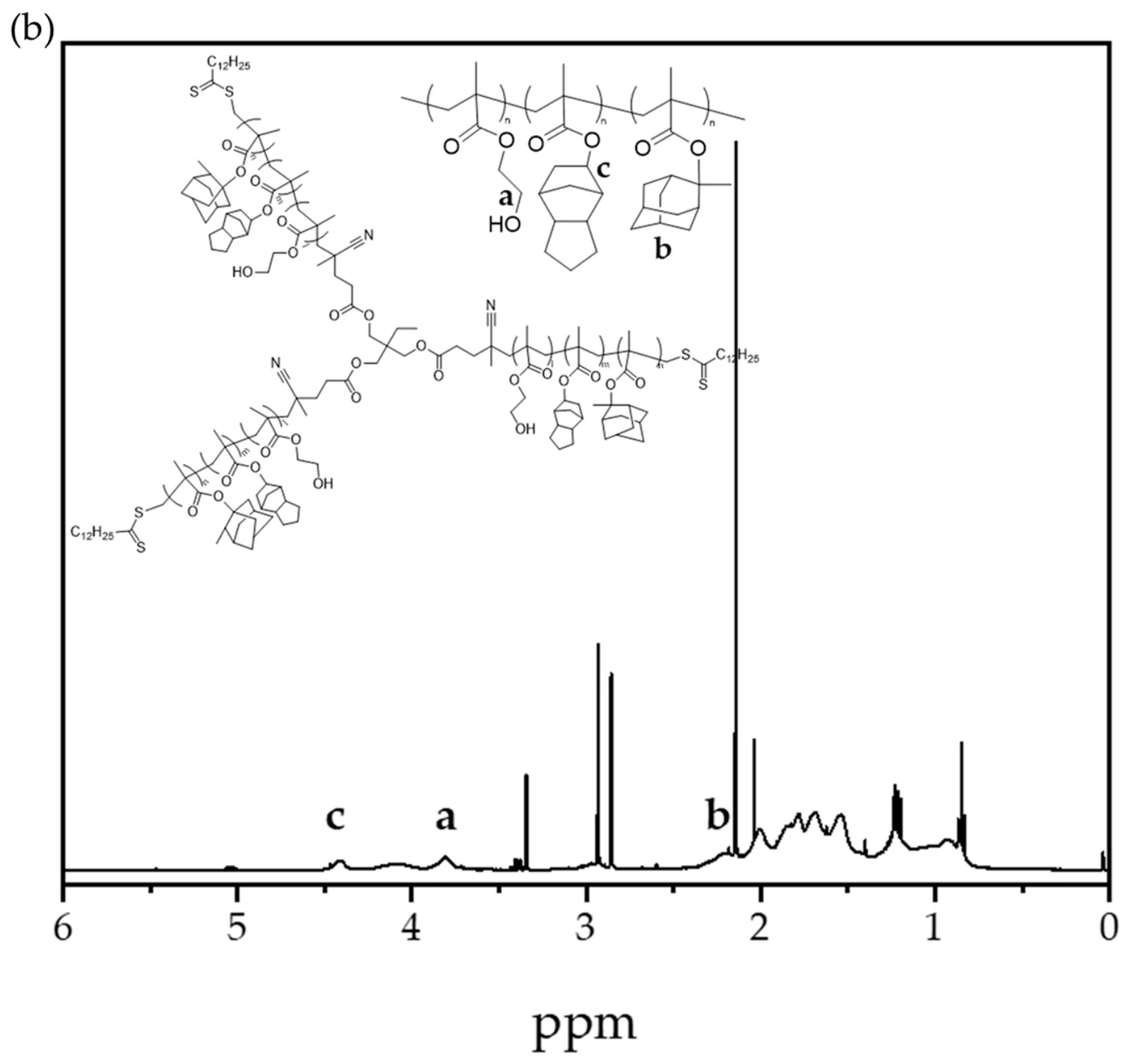
^1^H NMR spectra of the synthesized three-arm star polymers: (**a**) HTT 334 and (**b**) HTM 334. The inset structures explicitly depict the TMP-CEPA_3_-centered three-arm polymer architecture, and the enlarged representative arm segments are shown for peak assignment. The labels a–c denote the characteristic proton signals corresponding to the labeled protons in the inset structures.

To verify the chemical structure of the synthesized 3-arm CTA (TMP-CEPA_3_), ^1^H NMR spectroscopy was performed. The ^1^H NMR spectrum (Figure 1) indicated the 3-arm functionalization through integration ratios, consistent with the attachment of three CEPA arms to the central TMP core. Subsequently, the molar compositions of the synthesized 3-arm terpolymers were determined using ^1^H NMR spectroscopy (Figure 2). The initial monomer feed ratio for both polymers was fixed at HEMA:TCDMA:Protecting Group = 3:3:4. By integrating the characteristic proton peaks—such as the *tert*-butyl protons of *t*-BMA (δ = 1.43 ppm), the adamantyl ring protons of MAMA (δ = 1.50–2.20 ppm), the –OCH_2_– protons of HEMA, and the methyne proton of TCDMA—the incorporated monomer ratios were calculated [38,39,40]. As summarized in Table 1, the compositions were approximately 3:3.4:3.57 for HTT 334 and 3:3.3:4 for HTM 334. These results are consistent with the target feed ratio, indicating that the RAFT polymerization process allowed compositional control in the presence of the bulky MAMA monomers [27].

**Figure 3:**
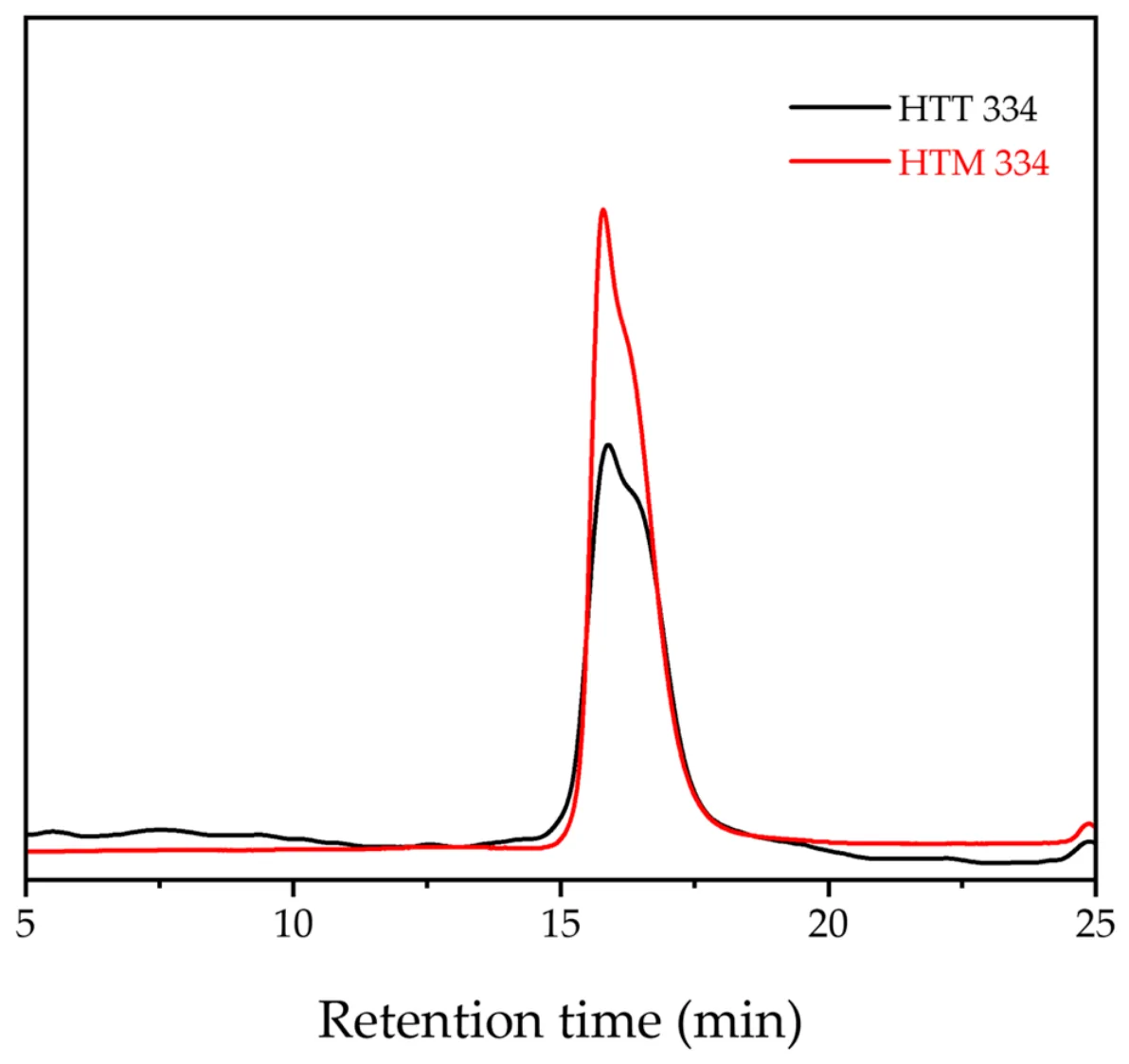
GPC traces of the synthesized HTT 334 (black line) and HTM 334 (red line).

In addition to the compositional analysis, the macromolecular characteristics of the polymers were evaluated using Gel Permeation Chromatography (GPC). As shown in Figure 3 and Table 1, HTT 334 and HTM 334 exhibited molecular weights of M_w_ = 36,087 g/mol and 36,775 g/mol, respectively, with narrow polydispersity indices (PDI) of 1.14 and 1.16. Obtaining a 3-branched architecture with a narrow PDI is relevant for minimizing intermolecular chain entanglement [7]. This structural characteristic is associated with more uniform dissolution during the NTI process with *n*-butyl acetate, which is a factor in reducing LER in patterning [15].

### 3.2. Thermal Properties and PEB Temperature Optimization

**Figure 4:**
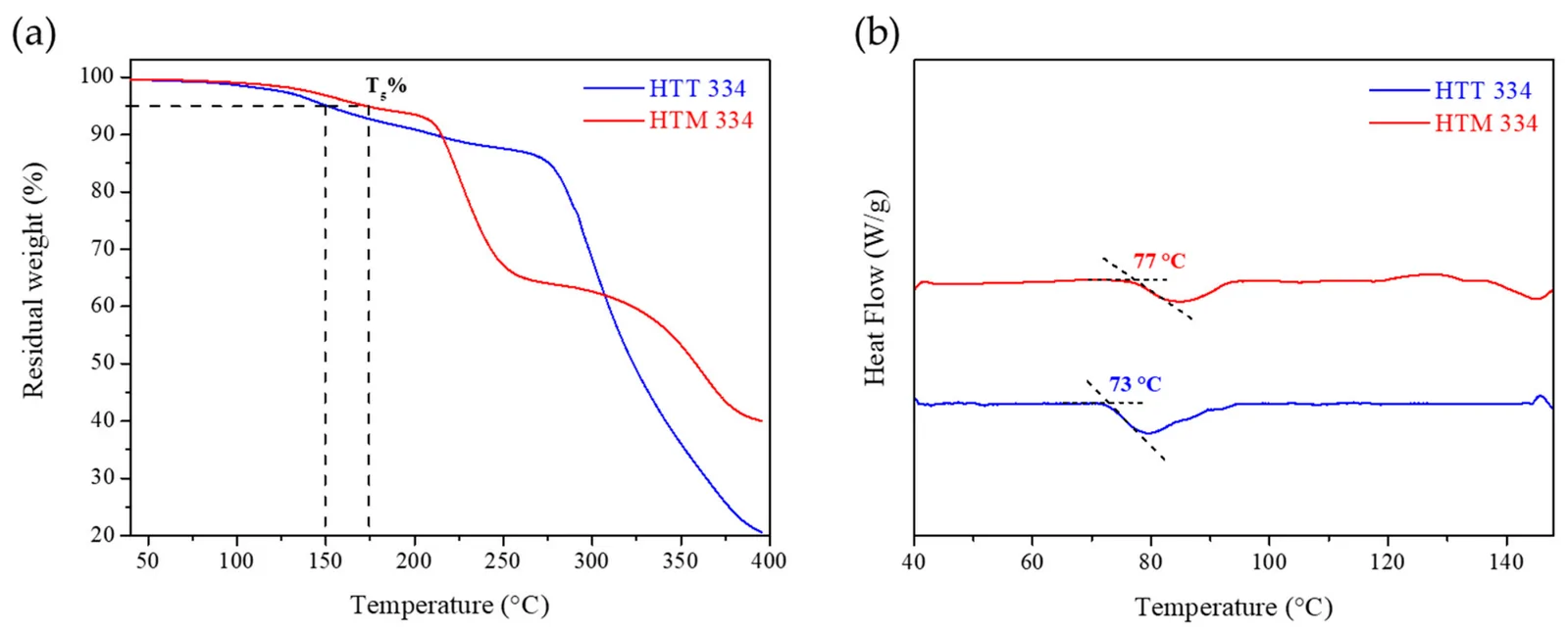
Thermal properties of the synthesized 3-arm polymers: (**a**) thermogravimetric analysis (TGA) curves showing the temperatures at 5% mass loss (T_5_%) of 151 °C for HTT 334 and 173 °C for HTM 334; (**b**) differential scanning calorimetry (DSC) thermograms indicating glass-transition temperatures (T*_g_*) of 73 °C for HTT 334 and 77 °C for HTM 334. The dashed lines in (**a**) indicate the 95% residual-weight level and the corresponding T_5_% temperatures, whereas the dashed lines in (**b**) indicate the glass-transition temperatures T*_g_*.

The thermal stability and deprotection behavior of the synthesized 3-arm photoresists were investigated using thermogravimetric analysis (TGA). To provide a consistent quantitative comparison, the temperature at 5% mass loss (T_5_%) was used as the characteristic thermal stability parameter. As shown in Figure 4a, the T_5_% values were 151 °C for HTT 334 and 173 °C for HTM 334, indicating greater initial thermal stability of HTM 334. The deprotection-related mass losses were approximately 12.1 wt% for HTT 334 and 28.7 wt% for HTM 334, which were close to the theoretical values of approximately 12.2 wt% and 28.9 wt%, respectively, calculated from the ^1^H NMR-derived copolymer compositions assuming complete cleavage of the corresponding acid-labile substituents. This agreement is consistent with near-complete removal of the acid-labile groups during the corresponding thermal events, although TGA alone does not directly identify the evolved species [40,41].

Following the evaluation of thermal degradation behavior, differential scanning calorimetry (DSC) measurements were performed to analyze the glass transition temperature (T*_g_*). These 3-arm star polymers can exhibit lower T*_g_* values compared to conventional linear polymers, which is considered a characteristic feature of branched architectures where an increased number of chain ends elevates the free volume [22,23]. In this study, the T*_g_* was primarily evaluated to establish a baseline for setting and optimizing the post-exposure bake (PEB) temperature around the T*_g_* temperature of each photoresist matrix [33]. As shown in Figure 4b, the T*_g_* values were determined to be 73 °C for HTT 334 and 77 °C for HTM 334. These measured thermal transitions provide the criteria for determining the appropriate PEB temperature window for both polymers during the negative-tone imaging (NTI) process.

### 3.3. FT-IR Spectral Changes After Exposure and Post-Exposure Baking

**Figure 5:**
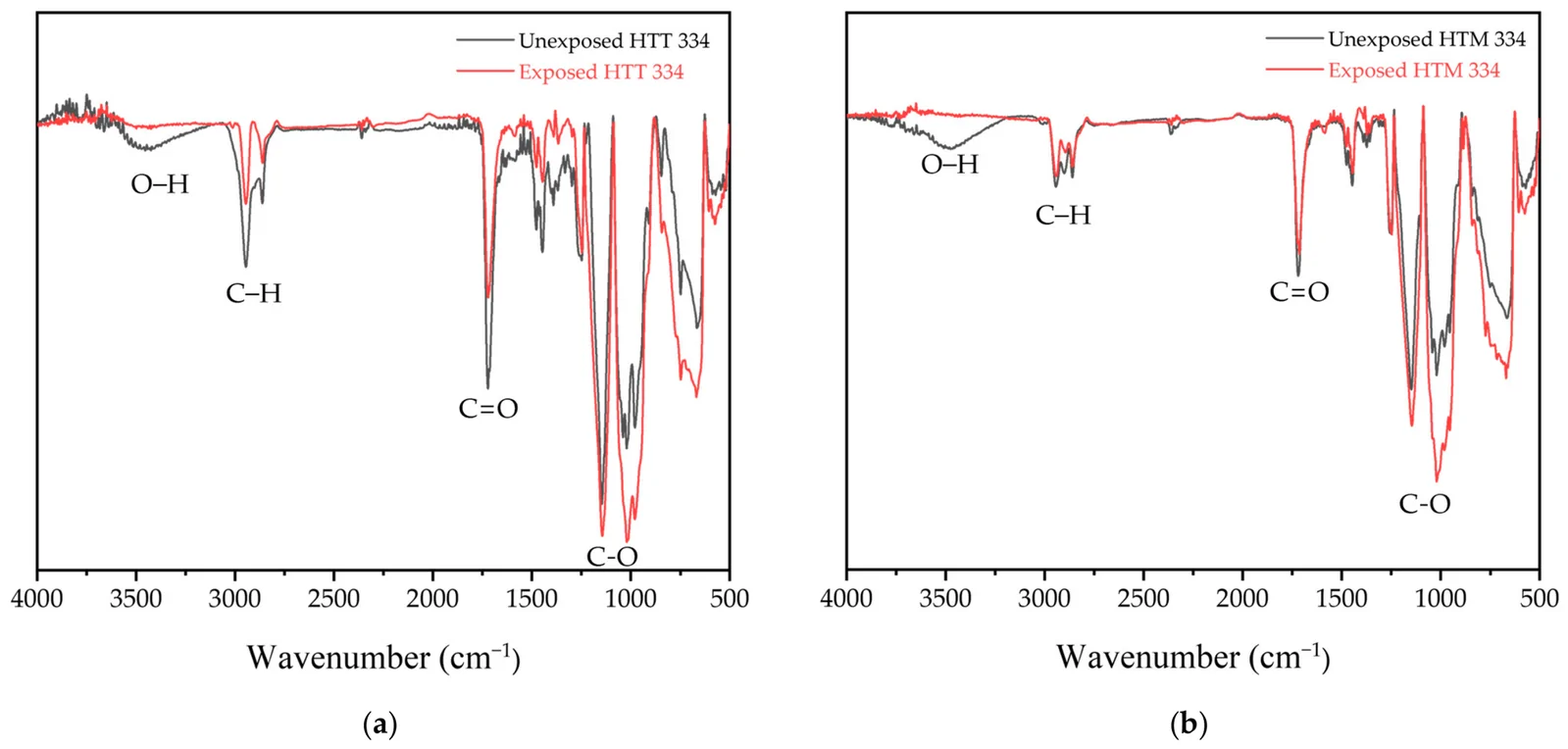
FT-IR spectra of the synthesized three-arm photoresist films before and after UV exposure and post-exposure baking: (**a**) HTT 334 and (**b**) HTM 334.

The negative-tone patterning of the synthesized three-arm photoresists is associated with exposure- and PEB-induced chemical changes that reduce the solubility of the exposed regions in *n*-butyl acetate [2,12]. Upon exposure at 365 nm, the photoacid generator (NDI) generates acidic species within the polymer matrix [13]. During the subsequent PEB process, the generated acid promotes cleavage of the acid-labile protecting groups, including the tert-butyl group in HTT 334 and the 2-methyl-2-adamantyl group in HTM 334, resulting in the formation of methacrylic acid-containing units and volatile deprotection byproducts [19,22]. These reactions increase the polarity of the exposed polymer regions and contribute to changes in dissolution behavior during organic solvent development. The corresponding FT-IR spectra of HTT 334 and HTM 334 before and after UV exposure and PEB are shown in Figure 5.

The generated carboxylic acid groups may also participate in intermolecular reactions with neighboring hydroxyl groups derived from HEMA units during PEB, potentially leading to reduced polymer solubility through intermolecular association or crosslinking-like behavior [36,42,43,44,45]. Although this transesterification-related mechanism has been proposed based on the chemical structure of the polymer, the present analysis does not independently confirm the exact reaction pathway or quantitatively determine the extent of intermolecular reactions [46,47].

In addition, considering that the synthesized polymers contain tri-functional trithiocarbonate groups derived from the RAFT chain transfer agent, photoinduced processes associated with RAFT-derived moieties may also contribute to the observed insolubilization behavior [29]. To investigate this possibility, PAG-free control experiments were performed using HTT 334 and HTM 334 under the optimized PEB conditions used for the PAG-containing resists (70 °C for HTT 334 and 90 °C for HTM 334). Interestingly, both PAG-free polymers exhibited clear negative-tone pattern formation after exposure, PEB, and development (Figure S2). These results suggest that photoinduced processes associated with the trithiocarbonate-containing RAFT architecture may contribute to the observed polymer insolubilization. This control experiment provides additional mechanistic insight beyond the conventional PAG-mediated deprotection pathway, because negative-tone pattern formation was observed even in the absence of PAG. Although the individual contributions cannot be quantitatively separated in the present study, this result suggests that the RAFT-derived polymer structure may also contribute to the overall pattern-formation process.

However, the present results do not independently distinguish the individual contributions of PAG-mediated acid reactions and RAFT-derived photoinduced processes. Therefore, the negative-tone pattern formation mechanism is considered to involve multiple exposure- and PEB-induced chemical transformations, including acid-mediated deprotection and possible RAFT-related photochemical reactions.

Quantitative evaluation of the deprotection extent by ^1^H NMR was also attempted using the exposed and PEB-treated films without development or washing. However, substantial overlap of the protecting-group resonances with the broad polymer signals in the 1–3 ppm region, particularly for HTM 334, prevented reliable quantitative integration. Therefore, the deprotection efficiency was not quantitatively determined by ^1^H NMR in this study.

### 3.4. Patterning Evaluation

**Figure 6:**
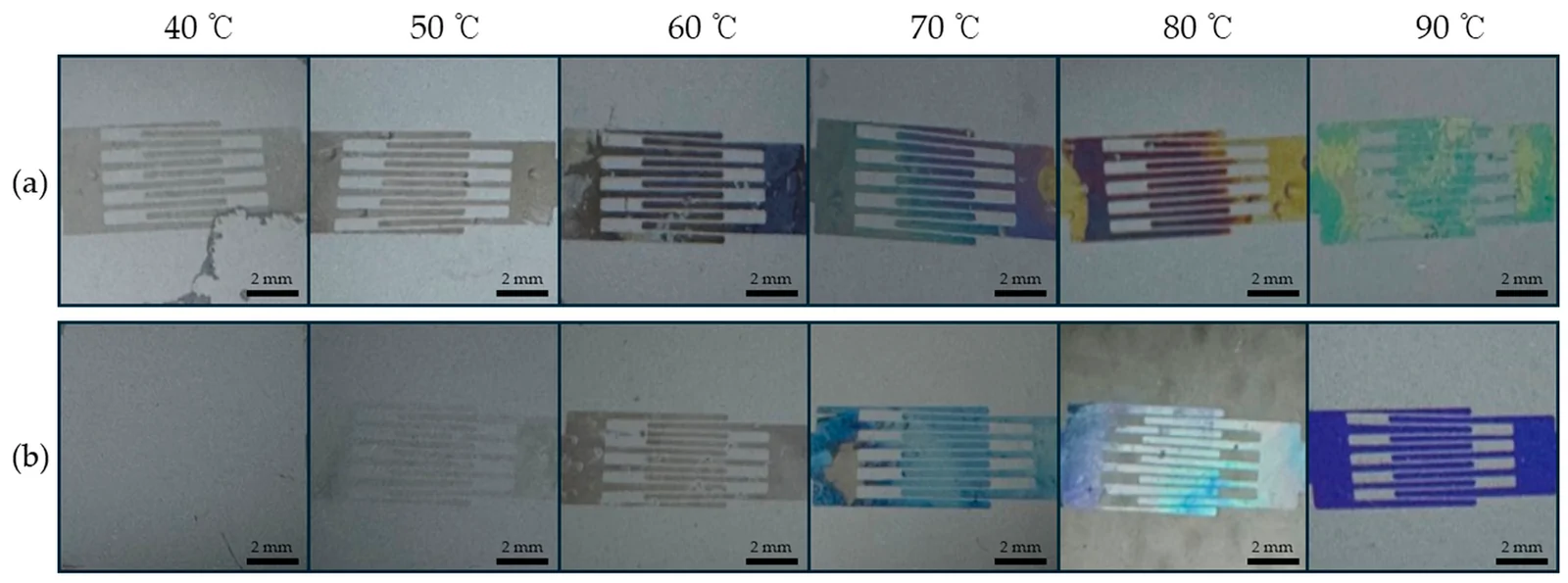
Optical microscope images of the patterned films obtained at different PEB temperatures from 40 to 90 °C: (**a**) HTT 334 and (**b**) HTM 334. The selected PEB conditions for stable pattern formation were determined to be 70 °C for HTT 334 and 90 °C for HTM 334.

The effects of post-exposure baking temperature on pattern formation were examined over the temperature range shown in Figure 6. Based on the optical microscope images, PEB temperatures of 70 °C for HTT 334 and 90 °C for HTM 334 were selected for the subsequent patterning evaluation. The difference between the selected PEB temperatures may reflect the combined effects of the thermal properties, deprotection behavior, and molecular mobility of the two polymer matrices [32,36]. Under these conditions, both photoresists produced distinguishable negative-tone line patterns after development in *n*-butyl acetate. Because photoacid diffusion was not directly measured, its individual contribution to the observed pattern formation could not be determined in the present study.

**Figure 7:**
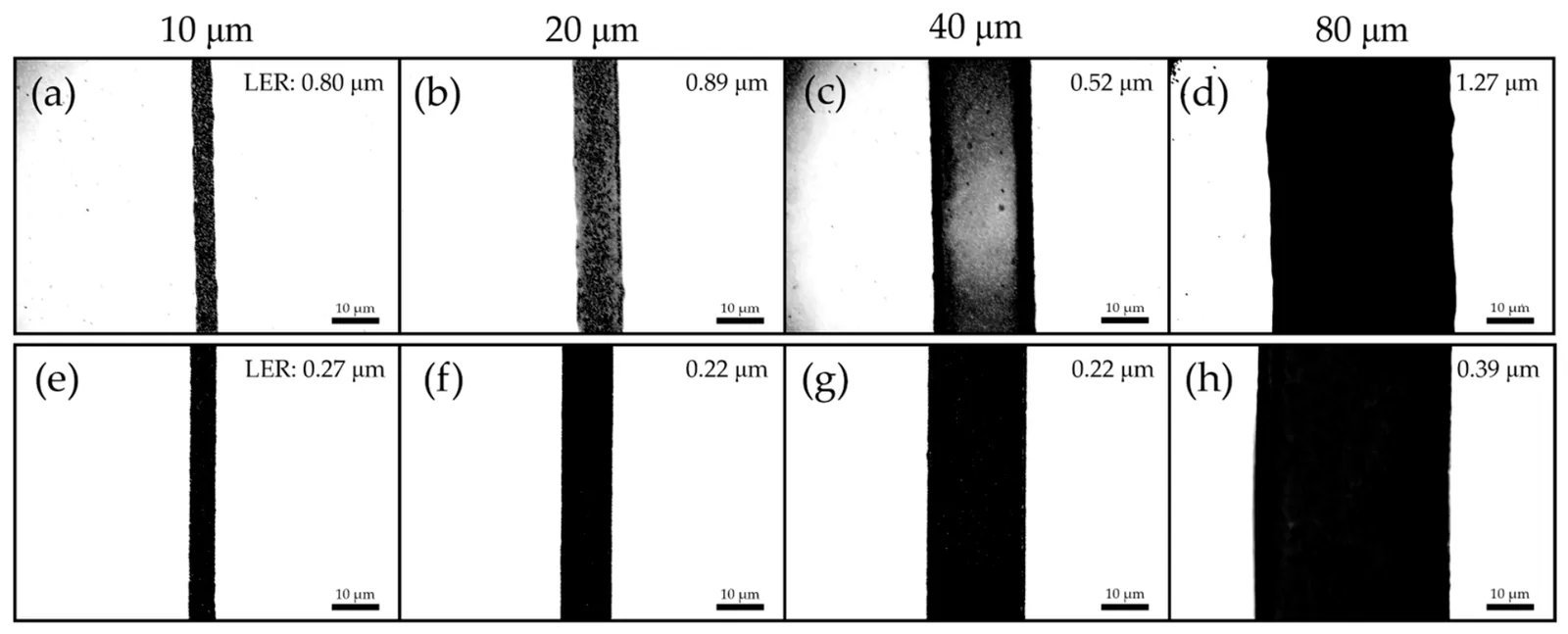
Optical microscope (OM) images of line patterns formed under the selected PEB conditions: (**a**–**d**) HTT 334 at mask dimensions of (**a**) 10 μm, (**b**) 20 μm, (**c**) 40 μm, and (**d**) 80 μm; (**e**–**h**) HTM 334 at mask dimensions of (**e**) 10 μm, (**f**) 20 μm, (**g**) 40 μm, and (**h**) 80 μm.

Using the selected PEB conditions, the pattern profiles of the two polymers were evaluated at nominal feature sizes of 80, 40, 20, and 10 μm. The corresponding optical microscope images are shown in Figure 7a–d for HTT 334 and Figure 7e–h for HTM 334. For HTT 334, increasing line deformation and edge irregularity were observed as the nominal feature size decreased to 10 μm. In contrast, HTM 334 retained more clearly defined line patterns at the nominal 10 μm dimension under the investigated conditions [17]. These differences may reflect the combined effects of protecting-group structure, deprotection kinetics, polymer mobility, and interaction with the *n*-butyl acetate developer. Because different PEB temperatures were used for the two materials, the effects of protecting-group structure and processing temperature cannot be fully separated based on the present results [7,22].

In addition to the qualitative observations from the imagery, a quantitative analysis of line-edge roughness (LER) was conducted to evaluate compliance with the reliability criteria, where LER is expected to be below 10% of the critical dimension (CD). In semiconductor manufacturing, managing LER is considered relevant since an LER exceeding 10% of the gate CD can induce variance in transistor performance [48]. As summarized in Table 2, the LER of the HTT 334 polymer at a ~10 μm CD was measured at 0.80 μm, resulting in an LER/CD ratio of 8.24%. On the other hand, the HTM 334 polymer showed an LER of 0.27 μm at the same CD, corresponding to an LER/CD ratio of 2.60%, which falls within the specified threshold.

**Figure 8:**
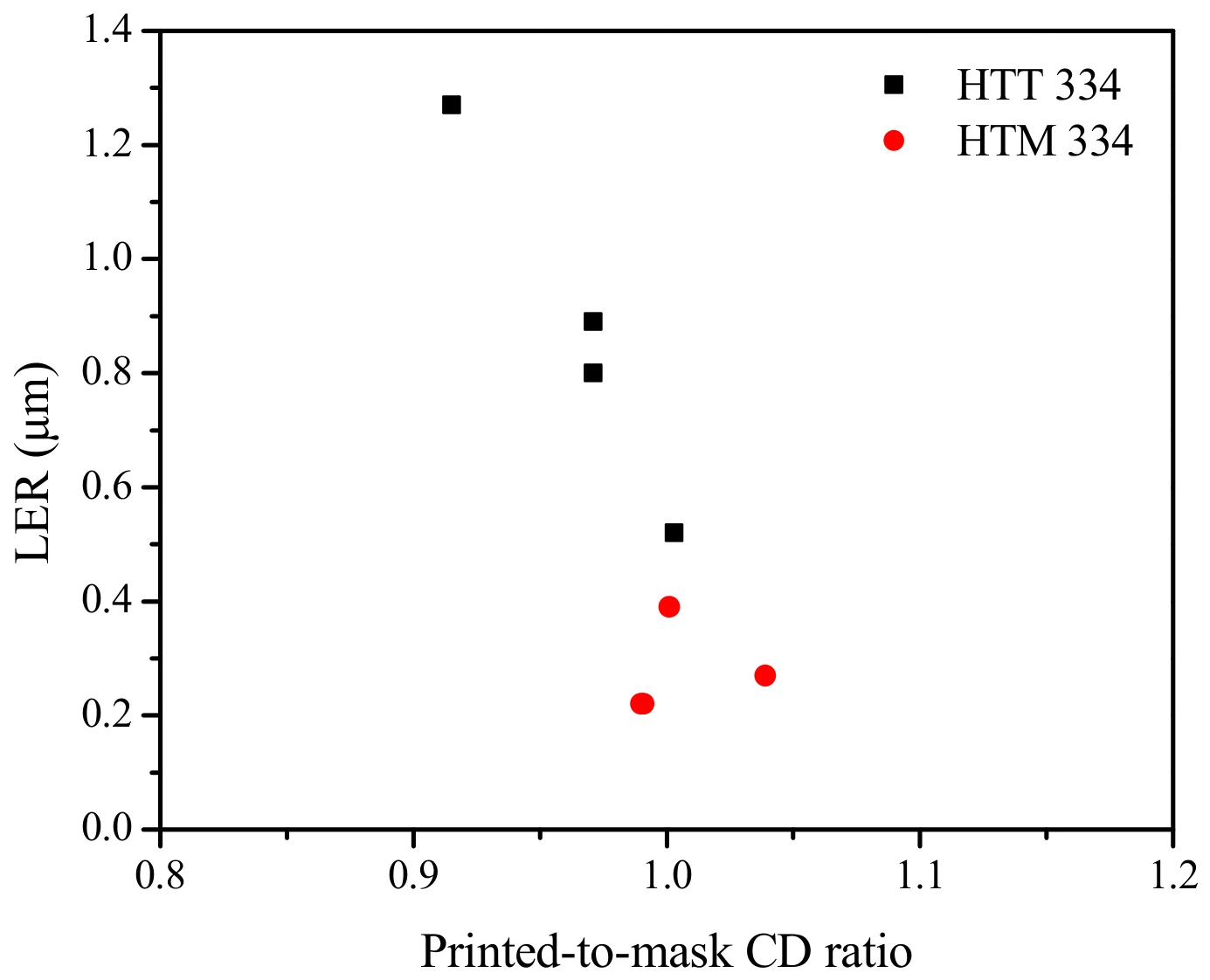
Evaluation of lithographic performance metrics: Correlation between printed-to-mask CD ratio and Line-Edge Roughness (LER) for the synthesized 3-arm photoresists.

To provide a broader evaluation of the resist characteristics, the relationship between the printed-to-mask CD ratio and LER was analyzed. While resolution is conventionally described by the physical half-pitch, the printed-to-mask CD ratio is also utilized as a metric for resolution in lithography. As feature sizes approach optical limits, the non-linear response of a photoresist can lead to an increase in printed-to-mask CD ratio, meaning that a printed-to-mask CD ratio value closer to the ideal target of 1 at a fine pitch indicates superior resolution capability. Mathematically, the printed-to-mask CD ratio represents the amplification of reticle CD errors onto the wafer, expressed as printed-to-mask CD ratio = ΔCD_wafer_/ΔCD_mask_ [4,49]. As shown in the printed-to-mask CD ratio-LER correlation plot (Figure 8), the lithographic quality is determined by how close the printed-to-mask CD ratio is to 1 and how close LER is to 0, where a simultaneous optimization toward these target values indicates superior patterning fidelity. The HTM 334 polymer displayed a highly selected distribution compared to the HTT 334 polymer, demonstrating a printed-to-mask CD ratio value tightly controlled near 1 and a significantly minimized LER approaching 0. These results indicate that, within the investigated three-arm polymer platform, the MAMA-containing HTM 334 exhibited more favorable printed-to-mask CD ratio and LER characteristics than the t-BMA-containing HTT 334 under the selected processing conditions.

## 4. Conclusions

In this study, three-arm star-shaped photoresists containing different acid-labile protecting groups were synthesized via core-first reversible addition–fragmentation chain-transfer (RAFT) polymerization to examine their thermal and negative-tone patterning behavior. HTT 334 and HTM 334, containing t-BMA and MAMA units, respectively, exhibited comparable molecular weights and narrow dispersities of 1.14 and 1.16. Thermogravimetric analysis showed T_5_% values of 151 °C for HTT 334 and 173 °C for HTM 334, while their glass-transition temperatures were 73 and 77 °C, respectively. Based on the observed pattern formation, PEB temperatures of 70 °C for HTT 334 and 90 °C for HTM 334 were selected for subsequent evaluation. FT-IR spectral changes after exposure and PEB were consistent with acid-catalyzed deprotection and possible intermolecular reactions involving the HEMA units, although the reaction pathway and extent of crosslinking were not independently quantified. Under the selected processing conditions, HTM 334 exhibited printed-to-mask critical-dimension ratios of 0.990–1.039 and measured LER values of 0.22–0.39 μm, whereas HTT 334 exhibited corresponding ratios of 0.915–1.003 and LER values of 0.52–1.27 μm. At the nominal 10 μm dimension, HTM 334 showed an LER of 0.27 μm and an LER/CD ratio of 2.60%. These results indicate that acid-labile protecting-group structure can influence thermal response and micrometer-scale pattern fidelity within the investigated three-arm RAFT polymer platform. The significance of this study lies in the direct comparison of two acid-labile protecting groups within a common three-arm RAFT photoresist platform, which enables the effect of protecting-group chemistry on patterning behavior to be evaluated with reduced influence from differences in overall polymer architecture. Furthermore, the PAG-free control experiments suggest that RAFT-derived photoinduced processes may contribute to negative-tone pattern formation in addition to the conventional PAG-mediated pathway. These findings provide further insight into the role of protecting-group chemistry and the possible contribution of RAFT-derived functionality in the design and patterning behavior of star-shaped photoresists.

## Figures and Tables

**Table 1:** Polymerization conditions, molecular weight characteristics (M_w_, PDI) determined by GPC, and monomer compositions determined by ^1^H NMR for HTT 334 and HTM 334.

Polymer	Molar Feed Ratios (Copolymer Composition)				M_w_ (g/mol)	PDI (*Đ*)
	HEMA	TCDMA	*t*-BMA	MAMA		
HTT 334	3 (3)	3 (3.4)	4 (3.57)		36,087	1.14
HTM 334	3 (3)	3 (3.3)		4 (4)	36,775	1.16

**Table 2:** Quantitative comparison of the critical dimension (CD), line-edge roughness (LER), and the LER/CD ratio for the HTT 334 and HTM 334 polymers at a ~10 μm resolution.

Polymer	CD, LER (μm)				~10 μm LER/CD (%)
	10 μm	20 μm	40 μm	80 μm	
HTT 334	9.71/0.80	19.42/0.89	40.1/0.52	73.16/1.27	8.24
HTM 334	10.39/0.27	19.81/0.22	39.61/0.22	80.11/0.39	2.60

## Data Availability

The original contributions presented in the study are included in the article/Supplementary Materials; further inquiries can be directed to the corresponding author.

## Supplementary Materials

The following supporting information can be downloaded at: https://www.mdpi.com/article/10.3390/polym18182223/s1, Figure S1. Schematic illustration of the negative-tone photolithography process used in this study. The exposed regions become insoluble after PEB and remain after organic solvent development, whereas the unexposed regions are removed during the development process; Figure S2. Optical microscopy images of negative-tone patterns obtained from PAG-free (a) HTT 334 after PEB at 70 °C and (b) HTM 334 after PEB at 90 °C under identical exposure and development conditions.
