# Supplementary material for: Effect of Acid-Labile Protecting-Group Structure on the Micrometer-Scale Patterning Performance of Core-First RAFT-Synthesized Three-Arm Photoresists
Source: Polymers (Basel). 2026 Sep 12;18(18):2223. doi: 10.3390/polym18182223 (PMC13611456; doi:10.3390/polym18182223)
Supplement: Supplementary file 1 [file polymers-18-02223-s001.zip › polymers-4504427-supplementary.pdf]

polymers

Supplementary Materials

Effect of Acid-Labile Protecting-Group Structure on the Micrometer-Scale Patterning Performance of Core-First RAFT-Synthesized Three-Arm Photoresists

Jinyoung Kim†, Yura Choi†, Eunsu Park, Kyeongjin Jang, Hyungjun Roh, and Namchul Cho*

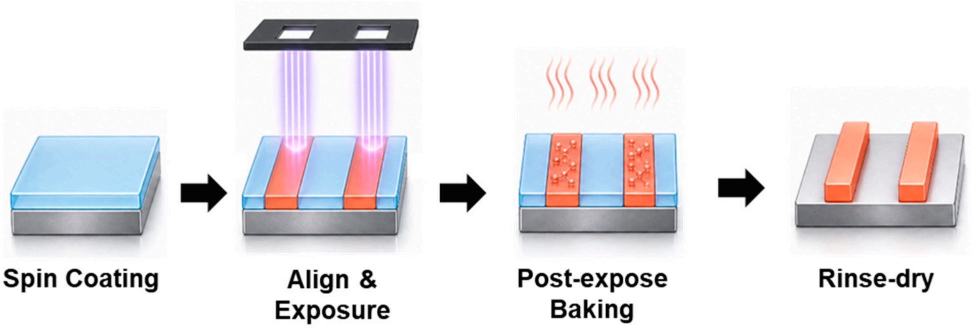

Figure S1. Schematic illustration of the negative-tone photolithography process used in this study. The exposed regions become insoluble after PEB and remain after organic solvent development, whereas the unexposed regions are removed during the development process.

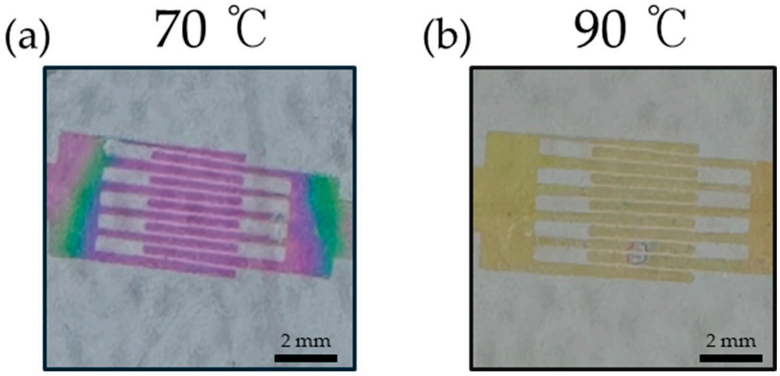

Figure S2. Optical microscopy images of negative-tone patterns obtained from PAG-free (a) HTT 334 after PEB at 70 °C and (b) HTM 334 after PEB at 90 °C under identical exposure and development conditions.
